# Efforts to mitigate the economic impact of the COVID-19 pandemic: potential entry points for neglected tropical diseases

**DOI:** 10.1186/s40249-020-00790-4

**Published:** 2021-01-04

**Authors:** John P. Ehrenberg, Jürg Utzinger, Gilberto Fontes, Eliana Maria Mauricio da Rocha, Nieves Ehrenberg, Xiao-Nong Zhou, Peter Steinmann

**Affiliations:** 1 Avenida Cedro 9, # 303, Cholul, Merida, Yucatan Mexico; 2 Retired from World Health Organization, Regional Office for the Western Pacific, Manila, The Philippines; 3 Swiss Tropical and Public Health Institute, Basel, Switzerland; 4 University of Basel, Basel, Switzerland; 5 Federal University of São João del Rei, Central-West Campus, Divinopolis, Minas Gerais Brazil; 6 International Foundation for Integrated Care, Oxford, UK; 7 National Institute of Parasitic Diseases at the Chinese Center for Disease Control and Prevention & Chinese Center for Tropical Diseases Research, Shanghai, People’s Republic of China; 8 School of Global Health, Chinese Center for Tropical Diseases Research-Shanghai Jiao Tong University School of Medicine, Shanghai, People’s Republic of China

**Keywords:** COVID-19, Economic recovery, Neglected tropical diseases, Scoping review

## Abstract

**Background:**

The damage inflicted by the coronavirus diseases 2019 (COVID-19) pandemic upon humanity is and will continue to be considerable. Unprecedented progress made in global health over the past 20 years has reverted and economic growth has already evaporated, giving rise to a global recession, the likes of which we may not have experienced since the Second World War. Our aim is to draw the attention of the neglected tropical disease (NTD) community towards some of the major emerging economic opportunities which are quickly appearing on the horizon as a result of COVID-19.

**Main text:**

This scoping review relied on a literature search comprised of a sample of articles, statements, and press releases on initiatives aimed at mitigating the impact of COVID-19, while supporting economic recovery. Of note, the donor scenario and economic development agendas are highly dynamic and expected to change rapidly as the COVID-19 pandemic unfolds, as are donor and lender priorities.

**Conclusions:**

The NTD community, particularly in low- and middle-income countries (LMICs), will need to work quickly, diligently, and in close collaboration with decision-makers and key stakeholders, across sectors at national and international level to secure its position. Doing so might enhance the odds of grasping potential opportunities to access some of the massive resources that are now available in the form of contributions from corporate foundations, trust funds, loans, debt relieve schemes, and other financial mechanisms, as part of the ongoing and future economic development agendas and public health priorities driven by the COVID-19 pandemic. This paper should serve as a starting point for the NTD community to seek much needed financial support in order to sustain and revitalize control and elimination efforts pertaining to NTDs in LMICs.
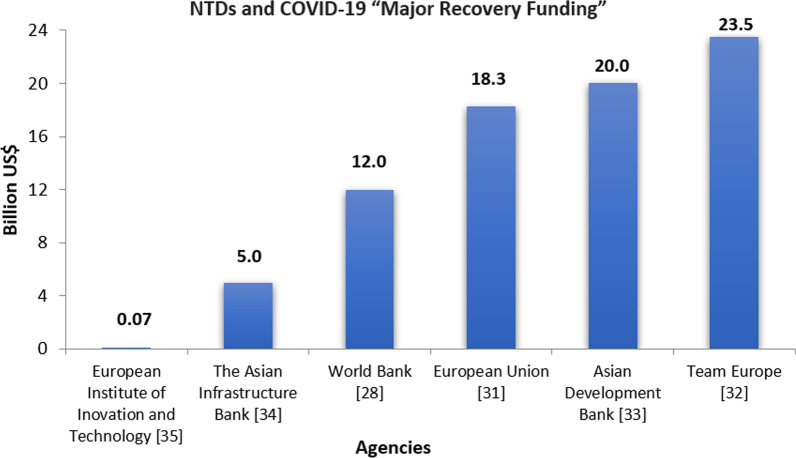

## Background

The emergence of a new disease cause by novel coronavirus—2019-nCoV, later named as severe acute respiratory syndrome coronavirus 2 (SARS-CoV-2) was observed in the People’s Republic of China in December 2019 [1]. On January 31, 2020, the World Health Organization (WHO) declared a global health emergency and, on March 11, 2020, coronavirus disease 2019 (COVID-19) was declared a pandemic, causing major impacts both in human health and societal activities [2]. In particular, the economic impacts of various containment measures began to ripple across the world and initial hopes for a swift recovery were soon dampened [3]. As difficult as it is to assess the magnitude of the collateral damage of the COVID-19 pandemic, there is no doubt that it was—and will continue to be—considerable. A global recession, perhaps of a magnitude not seen since the Second World War, is on the horizon.

In response, the international community has mobilized unprecedented economic recovery funds. At the time of preparing this piece for submission to the peer-reviewed literature in late September/early October 2020, the neglected tropical disease (NTD) community still struggled to reach out beyond a relatively small circle of established donors. Although integration of disease control and elimination efforts has become an accepted principle, there is room for broadening the scope well beyond the health and veterinary sectors. Our aim is to draw the attention of the NTD community towards some of the major emerging economic opportunities which are quickly appearing on the horizon as a result of COVID-19.

## Main text

The purpose of this paper is to provide a compass to NTD programme managers, researchers, decision-makers, and other stakeholders to navigate the rapidly evolving funding landscape through COVID-19 and beyond. It is based on a scoping review of the scientific literature, statements, and press releases on initiatives aimed at mitigating the impact of COVID-19, while supporting economic recovery. The scoping review entailed desktop research over a period of two weeks in August 2020 using the following key words: “COVID-19 and economic reactivation”, “major emergency funds for COVID-19”, “major funding for economic recovery and COVID-19”, “major funding for COVID-19”, “immediate funding for COVID-19”, and “major players supporting immediate economic recovery efforts and COVID-19”. Our search revealed the most important global and bilateral donors and lenders, their funding pledges and initiatives to support economic recovery and public health strengthening efforts.

**Fig. 1:**
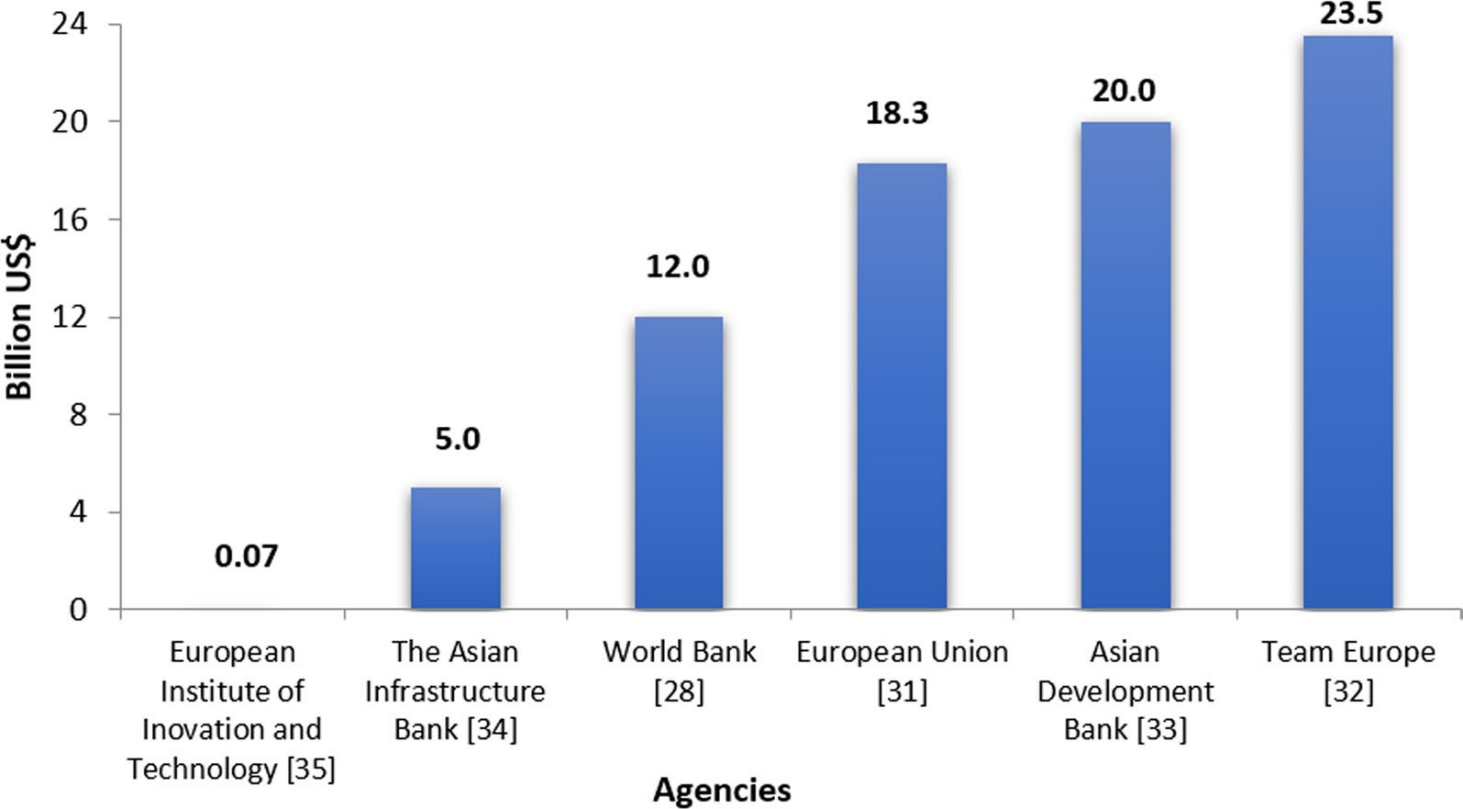
Examples of donors, pledges, and financial mechanisms to address the COVID-19 pandemic and economic recovery in USD billion

Some of the financial mechanisms ventured by a selection of the major league stakeholders are shown  in Table 1 and Fig. 1. The list is, by no means, exhaustive but might serve as a starting point for deeper investigations and consultations. Initiatives by the more traditional donors in the NTD field, such as the United States Agency for International Development (USAID), the Department for International Development (DFID) in the United Kingdom, the Bill & Melinda Gates Foundation (BMGF), and other philanthropic initiatives have been deliberately excluded from the review since these opportunities are generally well known in NTD circles.

**Table 1:** Potential points of entry for the neglected tropical disease (NTD) community to tap into current and planned future recovery and pandemic mitigation initiatives (scoping review pursued in August 2020)

Agency	Initiative(s)	References
World Bank	Accelerating India’s COVID-19 Social Protection Response Program Allocating USD 1 billion for the health sector	[26]
World Bank	USD 12 billion Fast Track Package for developing countries to strengthen health systems and bolster public health intervention	[28]
World Bank	Health Emergency Preparedness and Response Multi-Donor Fund (HEPRF) to provide incentives to low-income countries to increase investments in health preparedness and support the immediate COVID-19 response. Plight is ongoing with Japan expressing interest to become its founding donor	[29]
European Union	EU Global Response to COVID-19 targeting poorer and more vulnerable countries and people. EUR 15.6 billion allocated primarily but not exclusively to Africa. Focus is on three areas, two of which are directly in health and research. EUR 5.55 billion earmarked thus far	[31]
Asian Development Bank (ADB)	USD 20 billion package to address the needs of its developing member countries as they respond to COVID-19. Priorities combine health and economic measures	[33]
United Nations Development Program (UNDP)	USD 500 million covering three thematic areas, including health systems support (USD 150 million), inclusive and integrated crisis management and response (USD 250 million), and social and economic impact needs assessment and response (USD 100 million)	[30]
The Asian Infrastructure Investment Bank (AIIB)	USD 5 billion crisis recovery fund to support countries and businesses during the pandemic	[34]
Team Europe	EUR 20 billion package to combat the COVID-19 pandemic and its consequences. Global EU Response to COVID-19 supporting partner countries and fragile populations	[32]
European Institute of Innovation and Technology	EUR 60 million, of which EUR 9.85 million are designated for health. Calls for proposals for ventures and innovation projects for entrepreneurs from all 27 EU Members States, other non-member European countries, Israel and Turkey to support the launch of new innovation projects tackling COVID-19 related challenges as part of the ‘Pandemic Response Projects’	[35]
World Health Organization	WHO estimated cost of USD 675 million. Donors and contributors have since (as of 30 April 2020) committed around USD 320 million to WHO’s appeal for its’ COVID-19 preparedness and response plan	[14]

The overarching goal of this scoping review is to draw the attention of the NTD community towards some of the major emerging economic opportunities, which are quickly appearing on the horizon as a result of the unfolding COVID-19 pandemic. The window of opportunity to include the NTD agenda within these major global efforts might be short-lived, and if not seized promptly, there is a risk NTDs may be left behind. This means billions of people at risk of NTDs might not benefit from these massive economic recovery and health systems strengthening efforts.

## Direct and indirect impacts of COVID-19

Three equally important arguments are addressed in this section. First, the direct impact of economic crisis on impoverished communities already at risk of NTDs; second, the fact that weak health systems will be further weakened by COVID-19-related pressures; and third, the fact that NTD control and elimination gains may be lost due to interruption of NTD control activities. .

According to the World Bank, the world might suffer a 5.2% contraction in global gross domestic product (GDP) in 2020 [4], the deepest global recession in decades resulting in lower investments, erosion of human capital through lost work and schooling, and disruption of global trade and supply chains. Emerging markets and developing economies will contract by 2.5% in 2020. Latin America’s economy is predicted to contract by 7.2–7.6%, the highest contraction of any region of the world [4, 5].

Health systems in many low- and middle-income countries (LMICs) were already weak before the COVID-19 pandemic. They will continue to be massively challenged to the point of collapse across the world, affecting public health programmes for both infectious diseases and non-communicable diseases. Furthermore, there is a shortage of personal protective equipment (e.g. face masks) hitting LMICs hardest, supply chains are strained or interrupted (e.g. donated deworming drugs might not reach the countries in need in time), and campaigns for the control and elimination of NTDs are postponed or discontinued (e.g. closure of schools will prevent deworming campaigns through the education sector) [6–8]. Hence, the ongoing COVID-19 pandemic not only has direct, but also indirect impacts on NTDs and these are likely to revert progress made over the past 10–15 years [9, 10]. Mathematical modelling, such as that carried out by the NTD Modelling Consortium, can provide quantitative insights on how NTD programmes could be impacted by the delays [11]. The health, social, and economic consequences of the COVID-19 pandemic have and will continue to be most profound for the most vulnerable citizens communities, and countries. Existing inequities have led to large portions of the population without the necessary social and economic safety nets. Incidentally, the same socio-economic and environmental determinants govern the global distribution of NTDs. Countries with a large informal sector, including emerging economies, were among the hardest hit by COVID-19 (e.g. Argentina, Brazil, Columbia, India, Mexico, Peru, Russia, and South Africa).

Two cases in point are Brazil and Mexico: the sixth and tenth most populous countries of the world. According to the Johns Hopkins Coronavirus Resource Center, as of November 21, 2020, Brazil and Mexico occupy the third and eleventh place with regard to the total number of laboratory-confirmed SARS-CoV-2 infections with 6 020 164 and 1 025 969 cases, respectively [12].

More than 95% of new cases of visceral leishmaniasis, one of the high-burden NTDs reported to WHO, occurred in 10 countries, Brazil among them. Seroprevalence of *Entamoeba histolytica*, another NTD (unfortunately not included in WHO’s 20 NTDs), remains as high as 42% in some rural communities of Mexico [13].

The informal sector makes up an estimated one-third of the GDP and about 70% of the total employment in emerging market and developing economies [4]. Staying home is not an option for the vast majority of workers in the informal sector. Physical distancing and adherence to basic prevention measures are also not possible for populations living in densely packed slums with no access to water, sanitation, and hygiene (WASH) [8, 14].

In the context of this scoping review, it is important to mention that Brazil, Russia, India, China, and South Africa (BRICS countries) represent nearly 50% of the world’s population, 23% of GDP, 30% of the territory, and approximately 18% of trade [15, 16]. The BRICS countries happen to also account for more than 30% of the world's children at risk for intestinal worm infections [17], an important group of NTDs. Disease control programme managers in the BRICS countries, as well as other LMICs, most urgently need to work with decision-makers at national and regional levels to identify and secure new funding streams that emerged in response to the COVID-19 pandemic and might be leveraged for the control and elimination of NTDs.

## The NTDs in the face of COVID-19

The NTDs comprise a group of communicable diseases of bacterial, fungal, parasitic, and viral origin [18]. More than half of the world’s population are at risk of NTDs, while they currently infect almost 13% of the world population [19]. Like COVID-19, several NTDs are of zoonotic origin. Yet, as their name suggests, they continue to be left out from most of the affected countries’ public health agendas. Development Assistance Committee (DAC) countries and multilateral donors have largely ignored funding NTD control projects [20]. Impoverished populations throughout the world are commonly burdened by one or several NTDs, not to mention other communicable and non-communicable diseases, and now also COVID-19.

Although the situation has improved for many NTDs as a result of successful prevention, control, and elimination efforts, the global burden of the NTDs, as expressed in disability-adjusted life years (DALYs), was still estimated at a considerable 17.3 million DALYs in 2017 [21]. This essentially means that in spite of the achievements of some of the most successful NTD elimination and control programmes, and to mention only three, there are still 205 million people worldwide at risk of contracting onchocerciasis and 1.1 million people blinded by this disease. There are 120 million people worldwide with lymphatic filariasis, 40 million of which are incapacitated by the disease, and 1.5 billion people worldwide (close to 20% of the world’s population) infected with intestinal worms [22]. The NTDs thus represent an important burden for the affected population groups, especially if one considers that they are simultaneously affected by other infectious, vaccine preventable as well as nutrition-related diseases. COVID-19 is expected to render the lives of millions of people affected by NTDs even more precarious and the odds of sustaining NTD prevention, control, and elimination gains are rather slim with disease rebounds to be expected as a result of the diversion of funds and human resources to address the COVID-19 pandemic [23].

## Efforts towards mitigation of the COVID-19 pandemic and activation of the economy: opportunities for NTD control programmes

### Global efforts

The determinants and risk factors behind COVID-19 and countless other communicable and non-communicable diseases lie well beyond the purview of the health sector alone [24]. Possible synergies between COVID-19, non-health sector, and NTDs prevention and control programmes were explored in a previous piece, stressing the need for well-defined programmes that will set the stage for a multi-sectorial approach [23].

Many countries failed to respond effectively to COVID-19, a fact that calls for a transformation of their surveillance and public health response systems in a post-COVID-19 world. Thus, investments in such systems should top the list of priorities of the major development and economic recovery initiatives. However, health services and integrated disease surveillance-response systems will need to undergo profound changes in order to find more effective ways of coping with future emerging and re-emerging diseases, epidemics, and pandemics. NTD control programmes should actively participate in defining innovative integrated surveillance-response systems, as they cannot afford to be left behind yet again [25].

According to the World Bank, the immediate priority for policy-makers should be to address the health crisis and contain the short-term economic damage [26]. Preserving the financial sector will be key towards promoting recovery as a well-functioning financial system can help firms stay alive and ultimately retain jobs. Sustaining economic activity is expected to free up funds to support the health system.

**Fig. 2:**
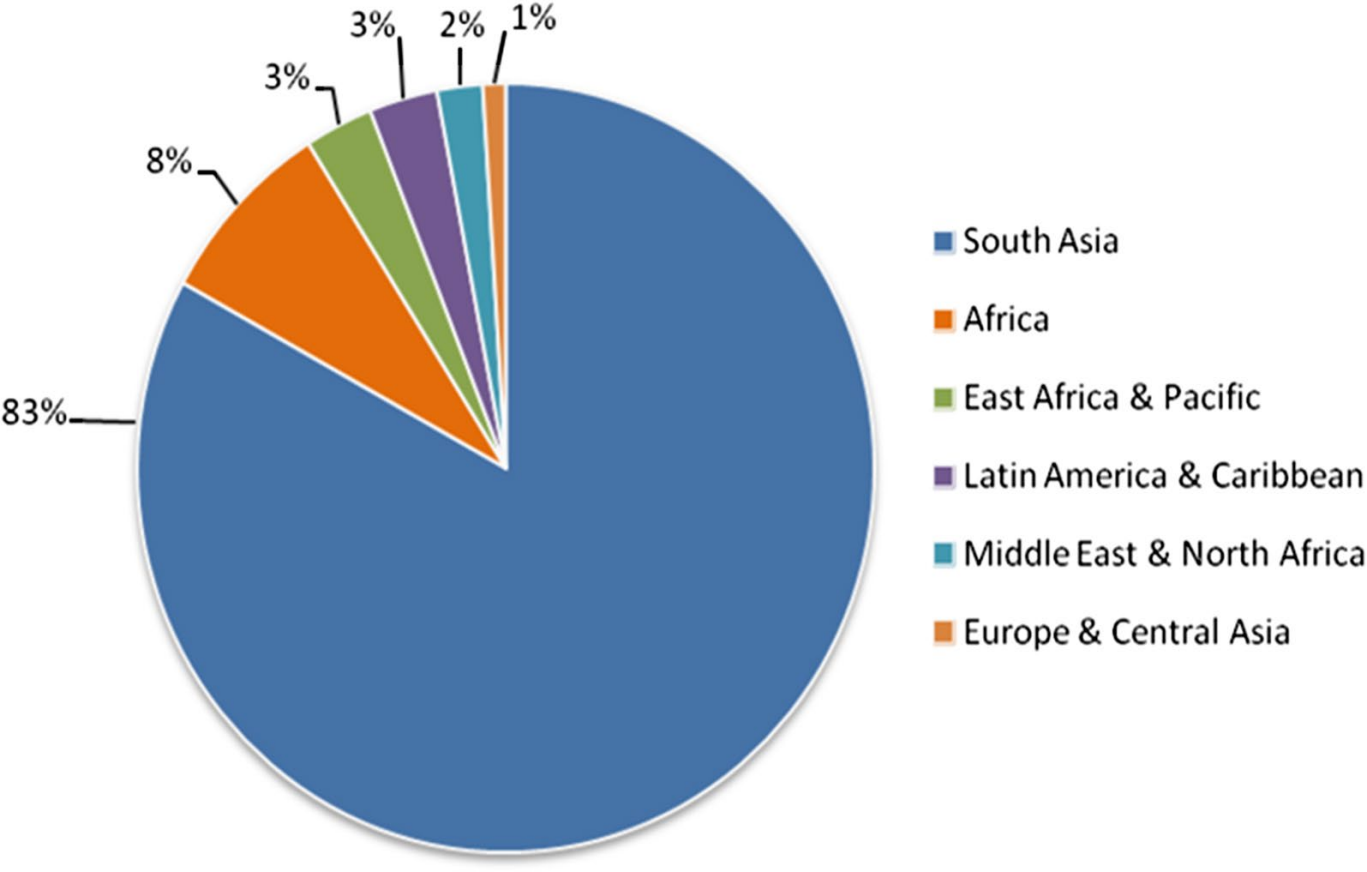
World Bank Fast Track Package COVID-19 response per region. Thus far, USD 1.5 billion have been earmarked [27]

**Fig. 3:**
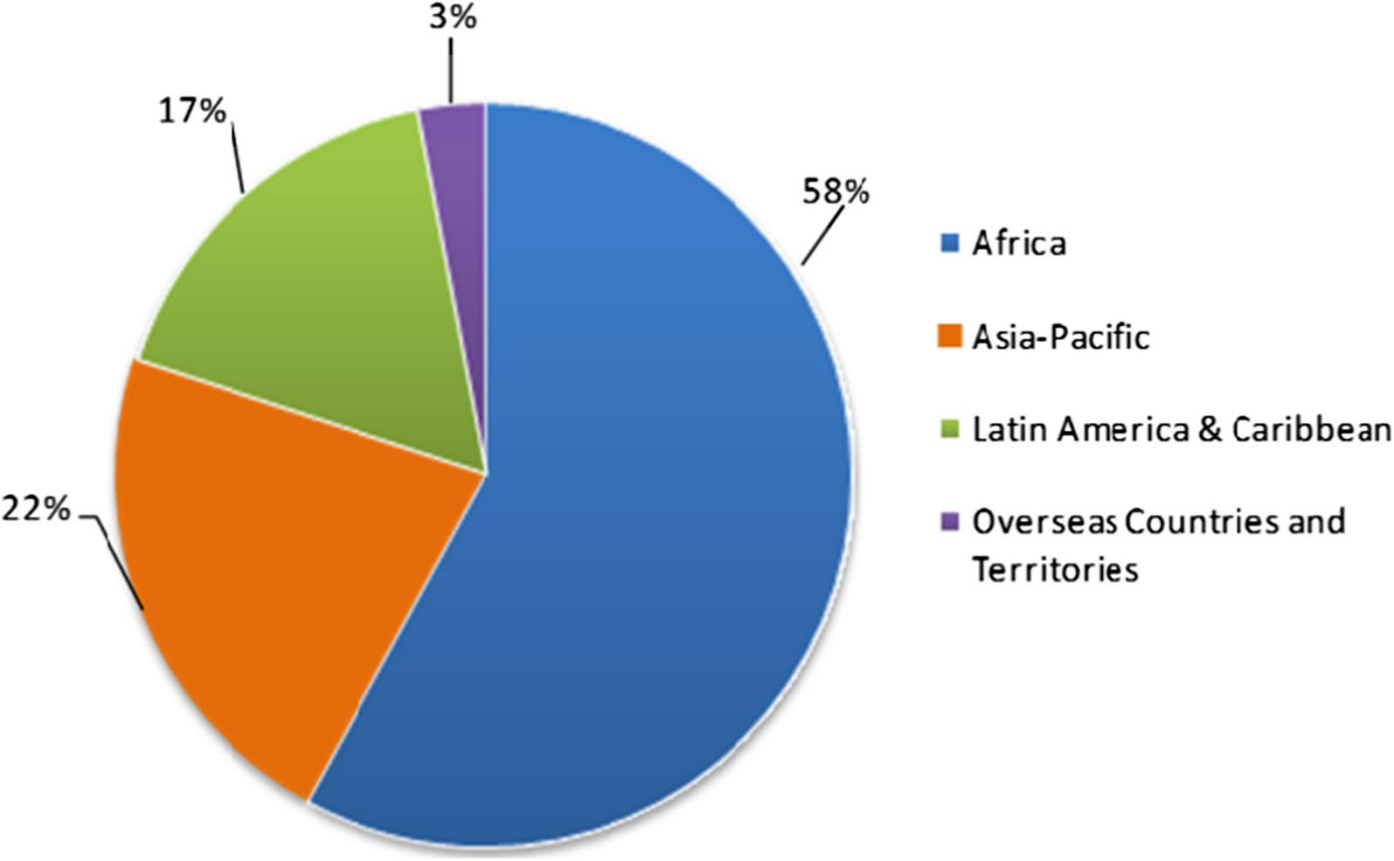
COVID-19 Global EU response per region. Current allocation of EUR 5.55 billion per region so far [31]

The World Bank committed early in the COVID-19 crisis to providing important additional financial resources for the world’s poorest countries. In a press release dated April 2, 2020 [27], the World Bank stated that it would be prepared to deploy up to USD 160 billion over the next 15 months to help countries respond to the COVID-19 pandemic and support economic recovery. A USD 12 billion fast-track package (in the form of low interest loans and grants) was announced to strengthen the COVID-19 response in LMICs and shorten the time to recovery [28] (Figs. 2, 3). Strengthening health systems is among this initiative’s top priorities. As part of this funding, interventions ranging from laboratory rehabilitation to equipping health centres with WASH infrastructure can be supported. Another World Bank effort is the Health Emergency Preparedness and Response Multi-Donor Fund (HEPRF). The objective of this umbrella funding scheme is to help countries develop strong public health capacity, including preparedness, disease surveillance, laboratory and diagnostic capacity, human resources, as well as emergency response operations [29]. The World Bank statement does not specify any amounts as it is waiting for pledges to be made by donor countries. Japan has already expressed its intention to become the founding donor of the new HEPRF [29].

The United Nations Development Program (UNDP) leads the UN’s socio-economic  response to the COVID-19 pandemic [30]. For its work, the agency relies on a network of over 3100 partners. A total of 52 countries are contributors to UNDP’s core budget in 2019. UNDP is working with over 50 governments across the world on Integrated National Financing Frameworks (INFFs) to align the COVID-19 response with the Sustainable Development Goals (SDGs). At the outset of the COVID-19 pandemic, in March 2020, UNDP presented a 3- to 6-month response budget of USD 500 million covering three thematic areas: (i) health systems support (USD 150 million); (ii) inclusive and integrated crisis management and response (USD 250 million); and (iii) social and economic impact needs assessment and response (USD 100 million). Whether donors and partners will live up to these expectations remains to be seen.

### European efforts

The European Union (EU) is the largest international donor, providing about 57% of the total global development assistance, while accounting for only a fifth of the global economy. The EU reacted swiftly to assist LMICs in their response to the COVID-19 pandemic [27], allocating EUR 15.6 billion with an emphasis on Africa (EUR 3.25 billion) (Fig. 3). Three priority areas have been identified: (i) emergency response; (ii) research, health, and water systems to combat the spread of coronavirus; and (iii) addressing the socio-economic  consequences of the COVID-19 crisis, including, in the longer-term, support for a recovery phase [31].

“Team Europe” is another EU response to COVID-19 supporting the most vulnerable and fragile populations in LMICs and conflict zones [32]. It targets primarily the informal sector of society, with a focus on Africa. Together, the European Commission, the European External Action Service, EU Member States, and financial institutions are launching a EUR 20 billion package to combat the COVID-19 pandemic and its consequences. The package combines resources from existing programmes (EUR 11 billion) with support from financial institutions such as the European Investment Bank and the European Bank for Reconstruction and Development (EUR 5 billion), and from EU Member States (EUR 4 billion).

### Asian efforts

The Asian Development Bank (ADB) announced a USD 20 billion package (in the form of loans, grants, and technical assistance) to address the needs of its LMIC members as they respond to COVID-19 [33]. Thus far, approximately USD 4.6 billion have been earmarked. Priorities include health and economic measures ranging from strengthening government’s alert and response capacities to addressing the COVID-19 pandemic’s economic and financial impact, and supporting various government measures targeted at poor people and vulnerable groups affected by COVID-19 through the loss of jobs and out-of-pocket health care expenditures. Eleven countries have already benefitted: Indonesia, the Philippines, and India are targeted to receive USD 1.5 billion each in sovereign projects [33].

The Asian Infrastructure Investment Bank (AIIB), of which the People’s Republic of China is the largest shareholder, created a USD 5 billion crisis recovery fund to support countries and businesses during the COVID-19 pandemic. With a recent capital injection, this programme can be tailored to respond to local needs [34].

### Other efforts

Other initiatives are considerably smaller but nevertheless relevant. A case in point is the European Institute of Innovation and Technology (EIT). It will make available EUR 60 million for entrepreneurs under the EIT Crisis Response Initiative to support 44 countries (Israel, Turkey, and 42 EU and Non-EU European countries) [35] in the launch of new innovation projects to tackle COVID-19 related challenges. A total of EUR 9.85 million are earmarked for health.

The Center for Global Development recently published an analysis on how international development agencies are responding to the COVID-19 crisis [14]. Included in the analysis is a WHO appeal for an estimated USD 675 million for a COVID-19 Preparedness and Response Plan, which saw donors pledge and commitment of around USD 320 million to date. The WHO also tracks partner funding and has already identified EUR 7.4 billion earmarked for COVID-19 response funding from 79 donors [36].

Development agencies are molding their aid packages according to their Government’s priorities. The German Federal Ministry for Economic Cooperation and Development (BMZ) is funding a EUR 1 billion emergency COVID-19 support programme targeting seven areas, with health and pandemic control heading the list (EUR 200 million) [37].

### How integration between health, social and community services could benefit NTDs

The speed and scale of the response required by the COVID-19 pandemic highlighted how the fragmentation in current health systems significantly impaired our ability to respond effectively in times of crises. Fragmentation leads to duplication, inefficiencies, poorer outcomes, and an unsatisfactory experience of care. There is growing evidence that integration of services in the health system and across sectors increases the resilience of systems [38, 39]. Until recently, integration efforts have tended to focus on improving coordination between primary and secondary care, or on strengthening relationships between health and social services. It is now widely recognized that social determinants, such as housing, education, employment, and social connectedness have a greater impact on health and well-being  than health and care services [40]. The focus is starting to shift towards integrating health and care with a much broader range of services, rooted in communities’ strengths, and needs. This is known as integrated community care (ICC). The importance of ICC is reflected in the WHO’s vision for primary health care that is based on three pillars: (i) an integrated health service delivery system; (ii) active community participation; and (iii) actions addressing broader social determinants of health [41].

The evidence shows integration works best when aimed at people with severe, complex, and long-term needs [42, 43]. It offers a new opportunity for managing morbidity and long term disabilities in the community, through greater coordination between health, social and community care. This is something that has not yet gained wide attention from the NTD community, and yet it may be worth exploring through further research. Perhaps even mass drug administration (MDA) campaigns related to some  NTDs would benefit from integration  with other activities beyond drug delivery [44]. Disease control programmes are part of complex health systems [45] and as such creating parallel funding, planning cycles, and additional reporting and data information systems need to be avoided [46].

ICC as a way to strengthen health systems and achieve Universal Health Coverage (UHC) would arguably make countries more resilient to shocks such as COVID-19, but whether these initiatives garner sufficient support from the main stakeholders and decision makers remains to be seen. This is particularly relevant in resource-constrained settings, which also harbour the very same highly vulnerable population groups most affected by NTDs [47]. NTDs may impose a considerable economic and social burden on individuals, families, and households, often related to loss of productivity but also abandonment of agricultural land due to morbidity, disability, and stigma [48, 49]. In addition, there are the direct costs of diagnosis and treatment and, even if diagnostics, drugs, and vaccines are offered free of charge, direct nonmedical costs such as transportation and accommodation which can easily add up to 20% of annual household income, propelling a previously stable household into an untenable debt [48]. It is ultimately the decision of the governments of endemic countries to make UHC including NTDs a domestic policy priority. To be effective, NTD control needs to be part of the national health plans and budgets and, ideally, also feature in those of other sectors [50].

## Conclusions and outlook

Funding NTD control and elimination is no longer business as usual, particularly in view of enhanced uncertainty, heightened volatility, and rapidly changing global health priorities in face of the unfolding COVID-19 pandemic. Innovative financing mechanisms have been suggested, such as development impact bonds, a form of “payment by results”, in which private investment is leveraged against commitments from governments and donors to pay for certain outcomes [50]. There are three key players in this novel funding mechanism: (i) “outcome funders”; (ii) private investors; and (iii) service providers or “delivery partners”. Private investors provide upfront funding to their delivery partners, who work towards measured outcomes. If results are delivered, the private investors are paid back by the outcome funders with an agreed financial return. Other innovative funding mechanisms might emerge in COVID-19 times. Whether they will turn out to be effective in sustaining and even up-scaling NTD control and elimination efforts remains to be seen.

Yet, there is no question that NTD control programme managers are facing a race against time to avoid further neglect [6, 23, 51]. Hence, they would be well advised to look into any possible new funding opportunities presented by the current COVID-19 pandemic. Some of the investments, such as integrated surveillance-response systems and improved WASH will directly benefit NTD control. Others might rather displace NTD funding. The necessary means to distribute free drugs to hundreds of millions of people every year in the frame of MDA campaigns will need to be secured, potentially from new donors as some traditional ones face economic hardship and increased competition for funding allocation to different public health issues. One thing is clear: donors and lenders have priorities and tend to target countries and recipients accordingly. The World Bank’s Fast Track Package has prioritized South Asia for its COVID-19 response, while the EU Global Response prioritized the African region (Figs. 2, 3). None of the two initiatives targeted Latin America and the Caribbean even though five of the world’s current COVID-19 hotspot countries—Brazil, Argentina, Columbia, Mexico, and Peru—are situated in Latin America (each of these countries reported more than 900 000 laboratory-confirmed SARS-CoV-2 cases as of November 21, 2020) [12]. It is conceivable that the financial needs of this region might be covered by other regional initiatives, such as the Inter-American Development Bank (IDB) at some point in the future. Clearly, there is room for commitments by other regional entities, including private foundations in the Americas (e.g. the Carlos Slim Foundation) [52]. Anti-corruption measures need to be taken into account to ensue transparency and to protect the public interests.

Many of the organizations and agencies mentioned in this paper have country offices. Every effort should be made by the NTD community, national and local authorities, academics, and non-governmental organizations to approach them and seek guidance on how these funds can be accessed and implemented most effectively. Key players should coordinate and partner at country level, preferably across sectors to increase their chances of funding. Some of the very same partners of major funding sources, such as UNDP or WHO have a cadre of advisors at country level with expertise in resource mobilization and often with access to funds and financial agencies. The current COVID-19 pandemic is a curse but it might also offer opportunities for the NTD community to jump on board some of the current major economic development initiatives targeting global economic recovery and mitigation of the pandemic.

It is the authors’ hope that this piece might help the NTD community revitalize itself at global and country level in order to sustain hard won programmatic gains now severely threatened by COVID-19.

## Data Availability

Not applicable.

## Abbreviations

ADB: Asian Development Bank
AIIB: Asian Infrastructure Investment Bank
BMZ: German Federal Ministry for Economic Cooperation and Development
BRICS: Brazil, Russia, India, China, South Africa
COVID 19: Coronavirus disease 2019
DALY: Disability-adjusted life year
DFID: Department for International Development
EIT: European Institute of Innovation and Technology
EU: European Union
GDP: Gross domestic product
HEPRF: Health Emergency Preparedness and Response Multi-Donor Fund
ICC: Integrated community care
IDB: Inter-American Development Bank
INFF: Integrated National Financing Framework
LMICs: Low- and middle-income countries
MDA: Mass drug administration
NTD: Neglected tropical disease
SARS: Severe acute respiratory syndrome
SARS-CoV-2: Severe acute respiratory syndrome coronavirus 2
SDGs: Sustainable Development Goals
UHC: Universal Health Coverage
UNDP: United Nations Development Program
USAID: United States Agency for International Development
WASH: Water, sanitation, and hygiene
WHO: World Health Organization
